# System models for resilience in gerontology: application to the COVID-19 pandemic

**DOI:** 10.1186/s12877-020-01965-2

**Published:** 2021-01-14

**Authors:** Katarzyna Klasa, Stephanie Galaitsi, Andrew Wister, Igor Linkov

**Affiliations:** 1 University of Michigan School of Public Health, Ann Arbor, USA; 2 United States Army Corps of Engineers, Engineering Research and Development Center, Vicksburg, USA; 3 Gerontology Research Centre, Simon Fraser University, Burnaby, Canada

## Abstract

The care needs for aging adults are increasing burdens on health systems around the world. Efforts minimizing risk to improve quality of life and aging have proven moderately successful, but acute shocks and chronic stressors to an individual’s systemic physical and cognitive functions may accelerate their inevitable degradations. A framework for resilience to the challenges associated with aging is required to complement on-going risk reduction policies, programs and interventions. Studies measuring resilience among the elderly at the individual level have not produced a standard methodology. Moreover, resilience measurements need to incorporate external structural and system-level factors that determine the resources that adults can access while recovering from aging-related adversities. We use the National Academies of Science conceptualization of resilience for natural disasters to frame resilience for aging adults. This enables development of a generalized theory of resilience for different individual and structural contexts and populations, including a specific application to the COVID-19 pandemic.

## Background

As improvements in health extend life expectancy worldwide, the chances of developing multiple chronic diseases by old age increase, as do the needs for complex care for people nearing the end of life [1–6]. Due to declining fertility and mortality rates, aging populations are burgeoning in relation to the younger cohorts who traditionally contribute to their care [7], causing societies to struggle to meet demands for increasingly complex care at higher costs with fewer resources [3, 8, 9]. Therefore, commensurate advances in illness prevention, adaptation, and coping are needed.

**Fig. 1:**
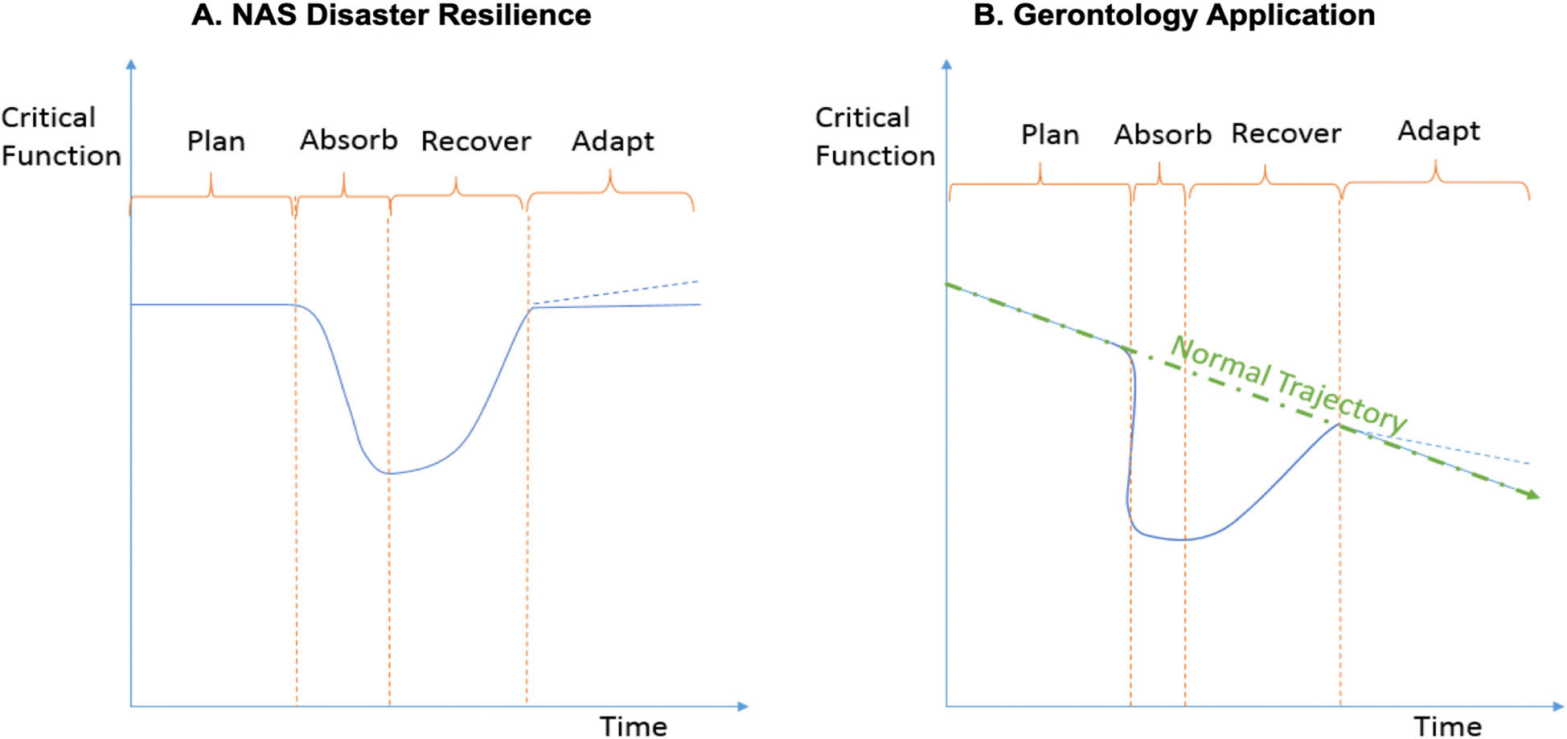
Applying National Academy of Science Resilience Model to Gerontology

Recently, academic scholars and health organizations have recognized the importance of resilience as a factor when modelling aging [10, 11]. The National Academies of Sciences (NAS) has defined resilience as “the ability to plan and prepare for, absorb, recover from, and adapt to adverse events.” [12] Under this definition, resilience is visualized as decline and recovery of critical functions following adverse events (Fig. 1) [13]. Resilience differs from risk and vulnerability by expanding the analysis to the processes that occur after a disruption is realized. Recovery, including adaptation, is an inherent part of resilience that materializes when certain risks and vulnerabilities cannot be wholly avoided because of their unpredictable nature or within feasible cost margins [14].

The NAS resilience model and its constituent phases (planning, absorbing, recovery, adaptation) were developed for disaster management but can be applied to older people experiencing adversity (i.e. the death of a partner) [15, 16]. Resilience for older adults comprises the ability to recover from disruptions, and some will recover better than others*.* Visually, critical functions trend down as the result of natural aging. Resilience allows individuals to cope with adverse events and effectively recover their critical functions (Fig. 1b). In contrast with paradigms predicated on an absence of or treatment of illness (or other debilitating challenges), a resilience perspective recognizes that adversity is a common experience and seeks to understand positive responses to disruptions. Early formulations of resilience were primarily psychological in nature, but recent advancements have expanded the concept’s scope to include individual and environmental domains, life course temporal dimensions, and applications to specific forms of adversity [17, 18].

Figure 1a visualizes the National Academy of Sciences (NAS) Disaster Resilience Model, highlighting the four constituent parts of resilience (i.e., planning, absorbing, recovery, adaptation). Figure 1b applies the NAS Disaster Resilience Model to Gerontology, visualizing aging on a trajectory of declining critical function over time. This figure was adapted after Linkov et al. (2014)

Different health fields show considerable diversity in the methods for modeling or quantifying resilience [19, 20]. We argue that the well-developed theory of disaster resilience can fill these gaps, turning resilience into a property of a system that can change depending on several parameters. Below, we review developments of resilience modelling in disaster and health literature, highlight existing gaps and difficulties in quantifying aging and resilience, and present a complex systems framework for quantifying resilience in older adults.

## Main text

### Resilience research developments and gaps

The first human explorations of resilience within psychological frameworks studied children, and later incorporated other stages of life (i.e. young adult, midlife, and finally the elderly) [21–23]. This trajectory influenced biomedical and pharmaceutical research to include resilience in theories of health trajectories and overall well-being during old age [24–26]. Some researchers have posited that “no generally accepted definition of resilience” exists [27]. While consensus over a common definition for resilience may never be reached across all health fields, there is agreement on its importance to health, and more specifically, gerontology [11, 21, 27, 28].

Current health-based literature indicates that resilience is conceptualized as either a mediator or a moderator in exposure-outcome relationships, deviating from the NAS definition [27–32]. For example, in physical domain, clinical studies have examined biomarkers such as musculoskeletal changes (adiposity, muscle mass, grip strength, bone mineral density, body weight, gait velocity), stem cell changes (% COP, COP Lamin A), serum markers (hemoglobin, albumin, oxidation products, antioxidants), metabolic markers (HbA1C), hormonal changes (DHEA, testosterone, Vitamin D), and new inflammatory markers (CRP, IL6, TNFa) [33–41]. Other studies have attempted to infer individual resilience by examining behaviors and subjective measurements such as emergency department visits, overnight hospital stays, and perceived pain. But these measure general health rather than an ability to absorb and recover from emerging disruptions [29]. Additionally, metrics used by researchers to quantify resilience do not always align with the outcomes that individuals deem important when adapting to a disruption in health (i.e. biomarkers and objective measures versus psychosocial factors and subjective measures) [42–44]. Better metrics are needed for new methodological approaches to coherently assess and model complex human systems.

Clinical studies have examined disease-specific resilience (i.e. Alzheimer’s Disease), focusing on neurobiological divergences or disease recovery (i.e. cognitive reserve, brain maintenance, frailty, function post-surgery) [45–49]. Over time, health fields have expanded aging theories, developing the idea of Successful Aging (SA) [10, 11, 50–52], But, SA and SA-based theories overlook common aging-related challenges that can disrupt health such as chronic illness. Additionally, SA-based theories often infer ‘failure’ if an elderly individual is not aging “successfully” according to a socially constructed definition. Resilience models of aging offer improvements over SA models in their ability to incorporate shocks and stressors beyond normal decline and to be tailored to an individual’s unique strengths and circumstances.

The World Health Organization’s (WHO) model of healthy ageing considers an individual as a product of their intrinsic capacity (i.e. personal characteristics, genetic inheritance, and health characteristics), extrinsic environmental characteristics, and functional ability (i.e. intrinsic capacity, extrinsic environmental characteristics and their interactions) [53–55]. Aging is positioned on a trajectory that entails three key periods (i.e. high and stable physical capacity, declining physical capacity, significant loss of physical capacity), within which physical capacity slowly declines as one grows older [53]. Here, resilience enables an individual to maintain high and stable functional ability and intrinsic capacity over their life time for as long as possible [51, 56, 57].

Unfortunately, a lack of consensus around operationalizing resilience has led to weak linkages between concepts and methods [15]. Fragmentation across disciplines has produced domain specific divisions of resilience such as physical, psychological, emotional, cognitive, health, motivational, community, cultural, spiritual, and creative resilience [22]. For example, social resilience is defined as “the ability of groups or communities to cope with external stresses and disturbances as a result of social, political and environmental change.” [58] Individual resilience is similar but focuses on the person instead of group. Community resilience differs, as it is framed as emerging from “a set of networked adaptive capacities” with dynamic attributes such as robustness, redundancy, and rapidity [59, 60]. It aligns more closely to public health definitions of community capacity which focus on not only the cultivation and transfer of knowledge but also community characteristics that affect “the ability to identify, mobilize, and address social and public health problems.” [59, 61] Thus, gerontology has encountered the same two obstacles that have inhibited resilience measurement in other complex systems: (1) resilience is often conflated with risk analysis and quantitative risk assessment, and (2) resilience knowledge is fragmented across disciplines that do not typically communicate with one another.

Polarized perceptions of old age further complicate matters. At one extreme, old age is viewed as an apocalyptic crisis of immense vulnerability, disengagement, and dependency, leading to a “care of the elderly” perspective. At the other extreme, old age is conceived as an important period of social engagement in which elderly contribute to all levels of society (e.g., capital generation, volunteerism, generativity, and intergenerational support), outweighing social costs with the benefits that they contribute. Neither perspective is wrong, but neither is entirely correct. An effective model of resilient aging requires a compromise between the two views.

**Fig. 2:**
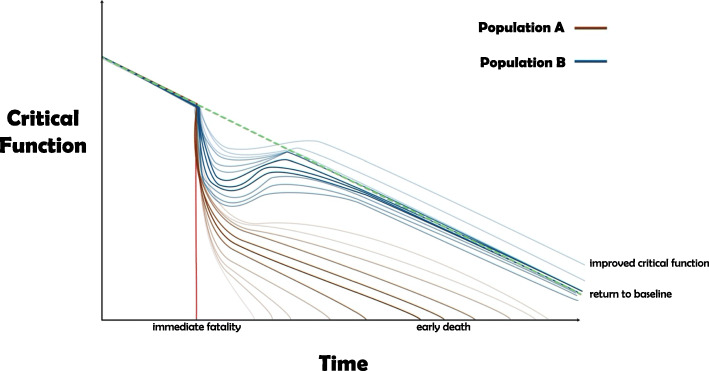
Population Resilience

A life course perspective can address both views and allows for some commonalities in conceptualizations of resilience [31]. The first commonality is that an individual faces some form of adversity over their life course. The second is that the individual has a positive response after facing adversity [62]. The third is that the goal of resilience is adaptation to adversity [10]. This adaptation may refer to establishing a newly optimal critical function operation, or to resisting the same pathway of downward degradation experienced by others in similar positions. In Fig. 2, we position aging on a trajectory where physical capacity slowly declines as one grows older. System shocks can precipitate regime changes and thresholds determine an individual’s ability to absorb a shock. Resilience supports an individual’s state of high and stable functional ability and intrinsic capacity over their lifetime for as long as possible.

Additionally, in Fig. 2, we show two populations: one comprised of resilient individuals that can recover and the other showing less resilient individuals who demonstrate the tendency of reduced critical functionality and earlier death. Resilience can therefore be understood in relative terms, such that an individual can be deemed more or less resilient than someone else. Defining threshold values that reflect transitions from one state to another (i.e. robustness, frailty, etc.) can help better inform decision-making about interventions in gerontology and geriatrics and at what point in the trajectory the interventions should be implemented for maximum efficacy and effectiveness. These threshold values can be personalized and retrofitted to an individual’s needs, goals, and outlook on life. Ultimately, degradation to a state of death remains inevitable, so resilience has limitations.

Figure 2 shows two populations of elderly individuals. Population B is comprised of resilient individuals that have the ability to recover, adapt, and return to baseline or close to baseline, with some even improving their critical function. Population A is comprised of less resilient individuals that demonstrate an inability to recover, adapt, and return to baseline, resulting in reduced critical function and earlier death. The blue and brown lines reflect a spectrum of possible trajectories for individuals that are part of Population B and Population A, respectively. The red line signifies a fatality that results in immediate death.

### Resilience framing and quantification in gerontology

Quantifying resilience for the elderly using a complex systems perspective can help indicate which sub-populations are better able to recover from disruptions and which populations merit either strengthened protection against disruptions or stronger support should disruptions occur. Resilience quantifications can also help planners manage disruptions, thereby allowing them to make resilience-informed decisions both during disruptions and in their absence to maximize long-term recovery or investments, respectively. However, there is no gold standard to measure or quantify resilience in aging, and studies are highly variable in definitions, measures and designs [63].

Existing conceptualizations of resilience and healthy aging link to observable and measurable outcomes, albeit inconsistent across studies. Researchers in health fields operationalize aging as a linear process (i.e. latent variable modelling and generalized mixed models), seeking to evaluate the effectiveness of clinical interventions [19, 29, 42, 64, 65]. While linear trends are useful for statistical analyses, aging is a complex, non-linear process that does not necessarily have a clear cause and effect relationship. Moreover, quantitative studies on resilience and aging are conducted using cross-sectional data [62], with few longitudinal studies existing despite their ability to provide greater insights into resilience across the lifespan. Additionally, benchmarks and thresholds are not consistent across studies. A complex systems perspective is necessary in order to address both upstream and downstream factors that impact life course resilience [13, 66–70].

**Fig. 3:**
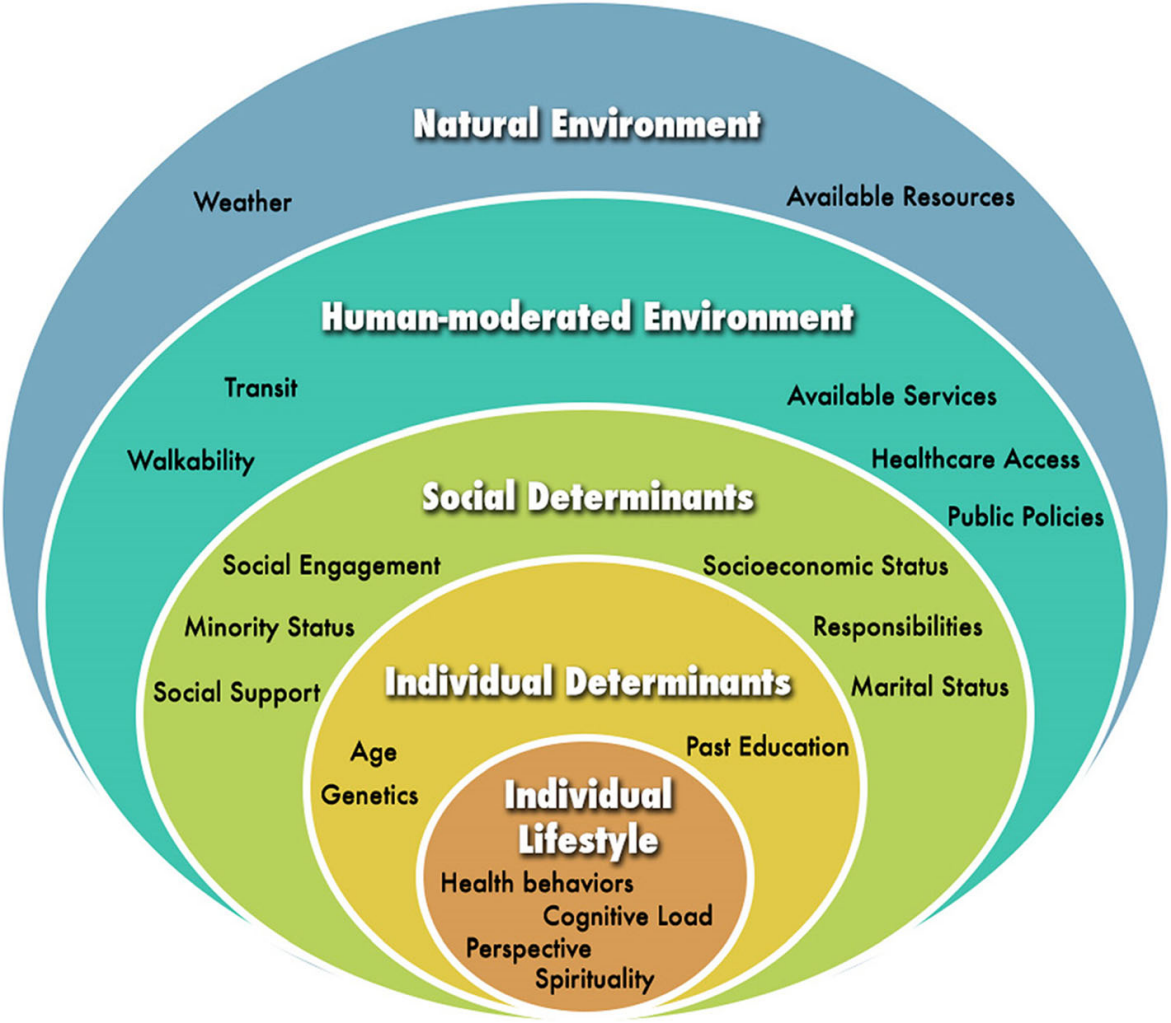
Socio-Ecological Model in Health

Complex systems illustrate the interdependent elements within a connected whole, where elements affect one another in subtle ways that can produce cascading effects [71–73]. Some studies have begun to examine complex system dynamics in aging by focusing on biological markers and physiological mechanisms of aging or on the general public health system [71, 74–76]. However, one model found in the aging and health literature that captures individual and external domains and can help identify inequities between the recovery capabilities of different populations is the socio-ecological model [77, 78] We propose adapting the socio-ecological model (Fig. 3) to a complex systems model for aging resilience, which would allow us to recognize individuals as nested within larger ecosystems and their embedded risks that are beyond their individual control [77, 78]. Furthermore, a complex systems model of resilience can provide quantifiable parameters that account for the different individual and environmental-level spheres of influence observed within existing socio-ecological frameworks. For instance, external factors such as poverty, societal perceptions of race, education, pandemics, and the physical environment can influence the health outcomes of a single individual. These factors play a larger role as time passes, meaning that they are of critical importance to the elderly [79–82]. Learning from social scientists, we can begin to incorporate these social determinants of health in quantitative models by using socio-economic, geographic information systems, social support, political, and demographic data [83–87].

Figure 3 visualizes the socio-ecological model and the five spheres (or levels) of influence (i.e., individual lifestyle, individual determinants, social determinants, human-moderated environment, and natural environment) on health behaviors and outcomes. Individuals are nested within a larger ecosystem and their embedded risks are influenced by factors within and outside of their control at each sphere of influence. This figure was adapted from McLeroy, Steckler, and Bibeau (1988).

Within the socio-ecological framework, five spheres influence an individual’s wellbeing, starting with characteristics of an individual, then expanding outwards to the larger environment. Applied to resilience in older adults, the first sphere embodies individual healthy behaviors such as social engagement and cognitive load. Next, the second sphere includes individual determinants that are outside the individual’s direct control, such as genetics, past education, and socio-economic status that affect how people may experience stressful events [88]. Third, we include the social realm, quantifying elements such as social cohesion and belonging [89–93]. Fourth is the built environment within which aging adults live, including aspects that support the nested systems, such as electricity, access to air conditioning during heat waves, greenspaces for physical activity, and walkability to cafes or grocery stores with fresh produce for healthy meals. Finally, there are changes in circumstances or resources of the natural world, such as pandemics, natural, meteorological, or human disasters [94–98].

These spheres directly impact numerous factors that determine health behaviors and outcomes, such as institutional factors, community factors, public policy (i.e. governance and law-making), intrapersonal factors, and interpersonal processes, and can be used to frame quantifications of individual resilience [99]. For example, recent public health “aging-in-place” [100–106] (supporting remaining in familiar environments as one ages) and age-friendly community [107–115] (environmental policies and practices to reduce barriers to active aging) efforts have attempted to use a broader systems perspective to support the long term resilience of aging adults by using a socio-ecological perspective [102].

### The resilience matrix

**Fig. 4:**
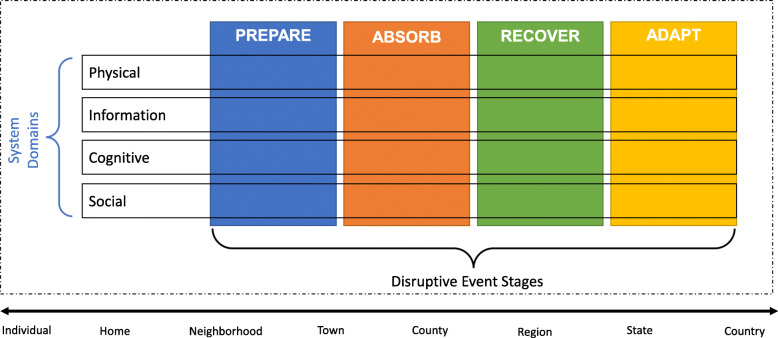
Resilience Matrix for Natural Disasters

The components of a resilience approach (preparing, absorbing, recovering, and adapting) encompass the different stages that medical and public health professionals employ for aging individuals and geriatric populations. The different spheres of the socio-ecological model can frame the scale of the resilience analysis. The resilience matrix (Fig. 4)—first developed by Linkov et al. (2013) and applied in different fields [68, 69, 96, 116]—combines the National Academies of Sciences system functions (plan/prepare, absorb, recover, adapt) with system domains (physical, information, cognitive, social), aligning with the socio-ecological model.

The matrix collects data in the physical realm, and translates it to information to be used for cognitive decision making [117]. These three systems domains encompass the first two spheres of the socio-ecological framework. A fourth systems domain (i.e. social) is overlaid on the socio-ecological social sphere comprising circumstances like reciprocity in social relationships, or social isolation. Thus, the resilience matrix examines resilience as instigated by the individual’s agency on an individual scale, while omitting the larger contextual factors. The matrix can be used for individuals evaluating their own resilience, while public health officials must navigate the implications of changes in the outermost spheres. For example, individuals and their doctors can target the component of the resilience matrix that emphasizes individual agency over various deterministic features of resilience and health systems. They may also recover from a disruption more effectively through improved formal-care and self-care processes according to the resilience matrix.

The Resilience Matrix shown in Fig. 4 combines the National Academies of Sciences (NAS) system functions and the Network-Centric Warfare domains. The rows of the matrix represent four systems domains that were adapted from the Network-Centric Operations doctrine in Alberts (2001). The columns represent the four stages of resilient systems from the NAS Disaster Resilience Model. The matrix can be applied to different scales, from micro (i.e. individual- or home-level) to macro (i.e. global- or country-level). This figure was adapted from Linkov et al. (2013).

The resilience matrix is constructed using existing research in health fields and by assessing their implications throughout the different stages and domains applied to a geriatric population. Each cell in the matrix include metrics that address the question: “How is the system’s ability to [plan/prepare for, absorb, recover from, adapt to] a health disruption among older individuals implemented in the [physical, information, cognitive, social] domain?” Since most aging metrics are difficult to measure through direct means, they must be estimated using a system-by-system basis that incorporates both quantitative and qualitative measures.

The relevant quantifications are dependent on the scale of the analysis and the scope of the objectives. Physical responses characterize the circumstances of an individual’s body. Informational responses encompass the information and resources available to individuals to help them cope during disruptions. Cognitive responses reflect the individual’s engagement with the changes needed during disruptions. Social responses encompass the existing structure of the individual’s social network and specifically its ability or willingness to support an individual, including circumstances in which the individual might not be actively engaged in seeking support [118]. Table 1 provides examples of these indicators within the Resilience Matrix. Each cell provides specific examples of indicators and metrics for each domain and stage of resilience that can be used to characterize and quantify resilience among older adults.

**Table 1:** Resilience metrics for older adults

	Prepare	Absorb	Recover	Adapt
Physical	Good state of health	Functioning systems available to respond	System works to restore lost function	Optimal value of lost function attained or improved upon
*Metric*	*Blood pressure, mobility, grip strength* etc.	*Immune system, other body attributes*	*Is recovery occurring, are system attributes improving?*	*Blood pressure, mobility, grip strength* etc.
Informational	Registered for relevant services and alerts	Identify problems, engage with appropriate agencies to resolve	Use the resources for needed support	Resource management
*Metric*	*Number and relevance of services signed up for*	*Does individual confront and address problems?*	*Output of resources according to disruption (money, assistance)*	*Do the resources meet the need over time?*
Cognitive	Awareness of baseline health and needs	Recognize new challenges and seek information and recommendations	Decision making and behavioral change to respond to new circumstances	Sustained behavioral changes
*Metric*	*Is individual aware of events? Does individual know baseline expectations for health?*	*Does individual recognize and act on emerging problems?*	*What behavior changes are committed to adjust to new circumstances?*	*Are the adaptive changes maintained over time?*
Social	Groups of friends and acquaintances	Social ties engage to ensure individual is reacting to disruption	Social ties provide resources and support	Social ties are retained despite new circumstances
*Metric*	*How many people does individual speak to in a week?*	*How many people contact individual in a week?*	*How many social ties able to provide support?*	*Percentage of ties independent of a specific context*

The resilience matrix in Table 1 can characterize resilience within individual control, although the socio-ecological model stresses that many factors influencing well-being are beyond individual control [119]. The stability and benefits of the outmost spheres of the socio-economic framework can reduce the burden of individual-level resilience. For example, a neighborhood with walkable streets and/or safe green spaces is more conducive to socializing among older people, which can foster social support and combat loneliness. Although resilience may be measured using an individual scale, the externalities of the socio-ecological model reveal opportunities to further enhance and anchor individual resilience within a broader coherent system. Ultimately, all individuals live within a community and are impacted by it, regardless of their level of engagement with it.

### Applying the resilience matrix to the COVID-19 pandemic

Coronavirus disease (COVID-19), caused by the SARS-CoV-2 virus, is highly contagious and associated with severe symptoms and high morbidity rates among older adults [120–130]. The virus has spread rapidly with multiple waves of infection, increasing to almost 40 million cases worldwide and more than 8 million in the U.S.; with over 1 million confirmed deaths globally and more than 218,000 in the U.S. [122, 131] Moreover, COVID-19 is increasingly conceptualized as a ‘gero-pandemic’—a disease that spread globally with heightened significance and deleterious consequences for older people [132–135]. The Centers for Disease Control and Prevention in the US show that, as of August 2020, persons aged 65–74, 75–84 and 85+ have both significantly higher probabilities of hospitalization and death than persons aged 18–24 (selected comparison group) [135].

#### Prepare

The pandemic has emphasized the importance of macro-level preparedness, and the integration of systems as a primary component of response to disasters that place older adults and other vulnerable groups at increased risk of mortality [128, 129, 136–138]. The resilience matrix can help individuals, localities and nations prepare for disasters such as pandemics [16]. For example, key factors that are critical to pandemic response can be identified for each system domain. Under the physical domain, individuals or policy makers can assess their baseline level of individual health or population health, respectively. Data on prevalence of comorbidities (by age and sex), quantity and geographic distribution of elderly or other vulnerable populations, a household’s accessibility to healthcare services, etc., can help establish a health risk index. Under the information domain, collecting data on capacities to educate the public about healthy behaviors and disease spread, to identify and limit misinformation and conspiracy theories, to contact trace, and to develop strong trust in government among the public is critical in ensuring that individuals understand the true risks of the disease to themselves and others. The cognitive domain includes metrics on capacities to disseminate transparent health communication and psychological resilience against the negative mental health consequences of a lockdown or quarantine. The social domain includes data on levels of community resilience and capacities for the public to safely engage with their social networks virtually and in person (i.e. ability to access internet, rates of mask adherence). Together, these can be combined to create a final preparedness index, allowing for more accurate forecasting and swifter response to a pandemic from the individual to the country-level.

#### Absorb

We have witnessed COVID-19 risk, resilience and response in both institutional and community environments across numerous countries around the world. However, older adults living in long-term care (LTC) (including congregate living environments, retirement homes, supportive housing, assisted living, etc.) are at the highest risk levels due to individual and system-level factors [139–143]. At the individual level, older people living in LTC tend to have very low resilience. Response and recovery to a pandemic, such as COVID-19, therefore requires attention to the structural system level and its intersection with individual risk [144]. Living in group quarters with group-based activities; congregate meals; high levels of frailty and cognitive impairment; having more severe and complex pre-existing conditions increase the disease risk and deleterious outcomes [145, 146]. Disease spread increases when staff are required to re-use personal protective equipment because of shortages, when staff work at more than one facility; and when training level is low. Andrew et al., (2020) point to the socio-environmental policy level, in order to understand system-level problems. These include COVID-19 testing ability, availability of personal protective equipment, resident and congregate room size, staff training in infectious disease, and synchronized administrative organization for mitigation strategies, all of which can be highly variable across health care jurisdictions and facilities [147]. There is also potential for applying or adapting communication technology that can be used by residents to stay connected with family and friends.

For example, the initial COVID-19 outbreak in the US occurred in Seattle WA, where several LTC facilities experienced rapid spread of the disease. The University of Washington Medicine’s (UWM’s) Post-Acute Care (PAC) Network put in place a coordinated three-phase approach in response to the pandemic in LTCs [148]. During the first phase of low COVID-19 cases, emphasis was placed on communicating response plans with all facilities; developing a systematic strategy for tracking cases; and preparing for distribution of personal protective equipment. During the second phase, at which point cases appeared, the response focused on education and training of staff and administration; implementation of testing criteria, supplies, and increased surveillance to identify potential cases; and the isolation of COVID-19 cases. The final third phase, during which time COVID-19 cases were spreading rapidly, a “drop-team” comprised of MDs, RNs and disease specialists was organized by the UWM and sent to targeted facilities. The drop team assessed and tested residents and staff; evaluated, triaged and organized transfer of patients to the Washington Disaster Medical Coordination Centre if needed; and notified local public health agencies [148]. Given the different socio-ecological contexts and resources across jurisdictions with different pandemic experiences, responses need to be retrofitted.

#### Recover

Examples of effective system-level responses to COVID-19 have begun to surface in the literature. The COVD-19 pandemic has revealed significant gaps in the long-term care system in most countries. At the socio-ecological level, Laxton et al. (2020) recommend several system-level LTC policy avenues during COVID-19. These include: collaboration across health care sectors; retrofitting approaches to the differential spread and clustering of the disease; federal direction in policy development and implementation in collaboration with other government jurisdictions; reforming the LTC regulatory system to adapt to a pandemic; reducing systemic inequalities in access to resources and treatment [140, 149].

Concurrently, public health should complement these efforts by providing access to and the ability to harness an umbrella of resources, such as healthcare services, safety, social support, and adequate education. Policies focusing on the environment should begin to remove barriers to participation that come with loss of function ability (i.e. age-friendly approaches), while concurrently providing avenues for compensating for such loss (i.e. affordable housing, innovative technologies for frail or disabled older adults, health education and promotion specialized transportation services). Once there is a significant loss of capacity, personalized long-term care services should be available and accessible. These services can support capacity-enhancing behaviors and ensure a dignified later life and ultimately a “good” death.

#### Adapt

Finally, the adapt phase will require careful assessment and evaluation of efficacy and effectiveness of programs and practices. This phase will directly feed into the prepare phase, as resilience is an adaptive and iterative process (see UWM example above).

## Conclusions

Aging is a dynamic process that occurs within a nested, complex system. Gerontological literature has hitherto emphasized the maintenance of health and reduction of risks rather than adaptation and recovery following a disruption. Risks to health and well-being over a person’s life course are unpredictable and difficult to quantify and model. Although the complexities surrounding risk as a concept have successfully been rendered into metrics-based approaches and models, there is currently no analogous framework for resilience. A complex systems model of resilience expands the focus beyond preventative care to care that is specific to recovery after unpreventable, random or normal aging disruptions. In examining the existing definitions of resilience for human health, we can better understand the motivations for supporting resilience, as well as the expected outcomes and auxiliary inputs needed or expected from the public sector. We apply our model to the COVID-19 global pandemic, given the speed and potency of its spread and pathogenic effects, coupled with the fact that it has affected older adults to a greater degree than other age groups.

Future models of aging should examine both quantity (number of years lived) and quality (wellness into old age). Resilience analyses should identify options for individuals to increase the likelihood of these positive eventualities, particularly following a disruption. These may overlap with risk management behaviors but will not be the same. For instance, healthy eating is typically framed as preventative medicine, leading to wellness. But, a person recovering from a heart attack with need to implement other distinct individual and systems changes, in which healthy eating will not be sufficient. While resilience is enhanced through the maintenance of wellness, its novelty as a concept is rooted in the positive and productive responses to wellness disruption. The health fields can thus benefit from a more explicit focus on recovery as in the NAS disaster perspective of resilience.

Our model provides a systems approach to complex processes that have multiple nested domains, emergent properties, and potential underlying processes. Even though this model only constitutes a Tier 1 approach in resilience analytics (Fox-Lent et al., 2018), it proves to be useful in understanding and managing the context of COVID-19 response and recovery. Early integration of resilience into the design of systems such as public health, community, and long-term care can help lay the groundwork for resilience thinking. Resilience requires the participation of the individual and their broader community; thus, it is useful to begin to view resilience as a property of an overall system [13]. Resilience is not solely an individual attribute or trait. To place the responsibility of resilience on an individual alone would remove the larger institutional contexts that also shape people’s health, and their access to resources necessary for recovery. It is inherently tied to the broader dynamic context including economic circumstances, physical surroundings, and positive social networks and relationships that may or may not relate to individual behavior, as well as the political supports made available to people lacking either economic security or long-term social support. These contexts depend on the decisions of government agencies, which are often charged with incompatible incentives, extreme partisanship, and uncoordinated federalism. Our hope is that the complex systems model of resilience and aging provides an integrative framework that can help facilitate both micro- and macro-level solutions to promote resilience over the life course.

## Data Availability

No data were used for this paper.

## Abbreviations

COVID-19: Coronavirus disease
NAS: National Academy of Sciences
COP: Circulating osteogenic precursor
Lamin A: Protein of the nuclear lamina
HbA1C: Glycated hemoglobin
DHEA: Dehydroepiandrosterone sulfate
CRP: C-reactive protein
IL-6: Interleukin 6
TNFa: Tumor necrosis factor alpha
SA: Successful Aging
WHO: World Health Organization
SARS-CoV-2: Severe acute respiratory syndrome coronavirus 2
U.S.: United States
UWM: University of Washington Medicine
